# Adhering to Social Distancing Rules Using a “Split Patient” Model With Rapid Cycle Deliberate Practice in Pediatric High-Fidelity Simulations

**DOI:** 10.7759/cureus.14117

**Published:** 2021-03-25

**Authors:** Rahul S Panesar, Erin Hulfish, Ilana Harwayne-Gidansky

**Affiliations:** 1 Pediatric Critical Care Medicine, Stony Brook Children's Hospital, Stony Brook, USA; 2 Pediatrics, Stony Brook Children's Hospital, Stony Brook, USA

**Keywords:** split, simulation, pediatrics, rapid cycle

## Abstract

The coronavirus disease 2019 (COVID-19) pandemic has required simulation-based medical education to adapt to physical distancing regulations in order to protect learners and facilitators. The “split patient” model allows for physical distancing of learners in pediatric high-fidelity simulations. This model was able to be used with the Rapid Cycle Deliberate Practice to teach pediatric residents basic and advanced life support skills and the principles of Crisis Resource Management.

## Introduction

Simulation-based medical education (SBME) has been a mainstream teaching tool for medical students and residents to augment their education and training [1,2]. SMBE has been further designed to provide trainees with hands-on experiences to learn skills and fill in knowledge gaps for high-stakes low-frequency events, such as cardiopulmonary arrest [3-5], especially in the field of pediatrics, where such events are even more rare than in adults [6]. These simulation sessions are usually conducted in a high-fidelity simulation environment, e.g., a simulation lab, or in situ to heighten the realism of the simulation scenario and potentially discover latent safety threats [7-9]. Our pediatric residency program provides both forms of SBME to trainees on a monthly basis. In our simulation lab, the Rapid Cycle Deliberate Practice (RCDP) method is used to optimize adult learning and skills practice with all participants [10].

Since the beginning of the coronavirus disease 2019 (COVID-19) pandemic, however, simulation education has needed to adapt to provide adequate safety measures to facilitators and students. Such changes have included wearing masks, physical distancing, and limiting crowds [11], and frequent hand disinfection. To comply with these recommendations and maintain infection-control safety, the number of learners in a simulation session was reduced in the simulation lab. Other SBME sessions have been changed to virtual tele-simulation sessions, all of which may compromise fidelity, conflict with the needs of learners, and worsen time constraints of facilitators. The struggle to balance education and infection control in the era of these restrictions has inspired further adaptation and innovation in our simulation program.

## Technical report

Methods

**Figure 1:**
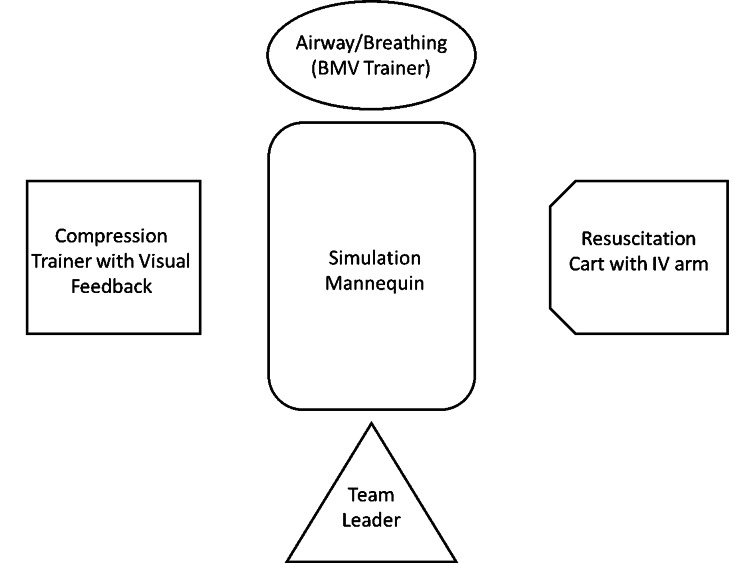
Split Patient Diagram in a Simulation Suite

In the spring of 2020, our institution implemented physical distancing rules due to COVID-19, placing a limit of four persons in the simulation lab, which conflicted with our traditional makeup of a six-person mock code team and left a void both in human resources during a simulation and educational resources for learners. Additionally, our pediatric simulation scenarios require participants to work closely on a high-fidelity mannequin, a practice which conflicted with the 6-ft physical distance rules. These restrictions hampered our ability to provide trainees the hands-on SMBE in our high-fidelity simulation suites. We consequently devised a new operating model to meet social distancing and educational goals, which called for “splitting” the simulated patient into modular work stations (Figure 1). 

**Figure 2:**
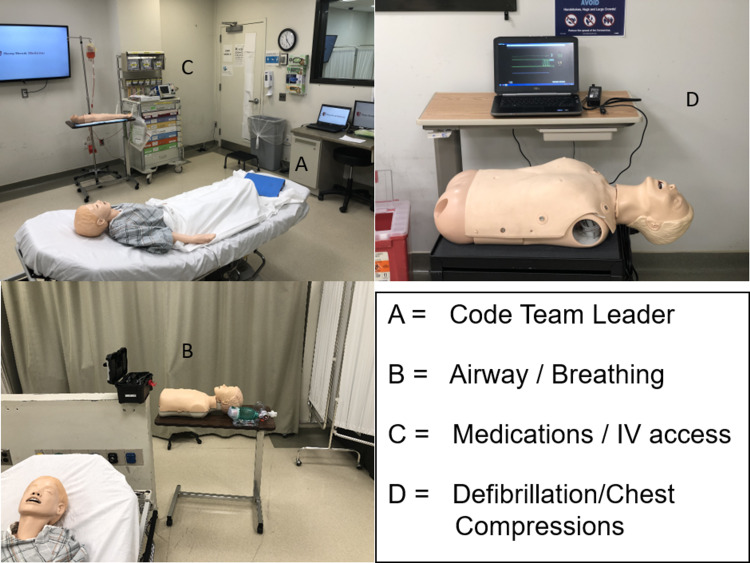
Positions of Team Members in Split Patient Simulation

Our high-fidelity simulation lab space is 144 square feet, connected with video and audio play-back to the control room, and separated by a two-way mirror. We positioned our mannequin (SimMan 3G, SimJunior, SimBaby, Laerdal Medical, Wappingers Falls, NY, USA) in the middle with separate work stations greater than 6 feet apart so that participants could be physically distanced. The stations included a simulation airway trainer with a bag-valve mask (BMV) kit at the head of the bed near the head of the mannequin; a separate simulation trainer for chest compressions with a video-play back software that would display depth and frequency of compressions (B-Line Medical, Washington, DC, USA), noting optimal technique, along with chest pads for defibrillation; an IV arm near the code cart with a working port so that the participant could draw and administer medications, and, finally, a position for the code team leader at the foot of the bed, where she/he could keep a “birds-eye” view on the team, monitor, and patient (Figure 2).

Participants were screened with a non-contact infrared thermometer and health questionnaire prior to entering the simulation center and were required to wear face masks and use hand sanitizer prior to being seated in a classroom. There they received the usual pre-briefing of the simulation, including educational goals and directives, the RCDP method, features of the simulation suite, and an explanation of the new “split patient”. They were then escorted into the simulation suite wherein they would begin their scenario, acting as the rapid response team (RRT).

A vignette was provided in a paper chart at the bedside with vitals displayed on a monitor screen near the head of the bed. The code team leader would position themselves at the foot of the bed, and one team member would take the paper chart and step away to read the vignette while other team members would move to their stations. After the team leader read the vignette out loud, participants moved to their station. Each team member was responsible for their respective resuscitation role, e.g., code leader, airway/breathing, circulation/chest compressions, and IV medications. Using the RCDP training method, the simulation would be stopped after several salient teaching points were reached, at the discretion of the facilitator, who was in the control room. These points were then discussed with the facilitator at the door of the simulation suite, maintaining physical distancing and the four-person limit in the suite. The participants were then escorted back to the adjacent classroom and the room was reset for a repeat of the scenario and rerun until enough teaching points were reached. We used Basic Life Support (BLS) and Pediatric Advanced Life Support (PALS) [12] as references to reinforce airway, breathing, and circulation skills, as well as Crisis Resource Management (CRM) principles for effective team coordination [13]. This process was repeated, usually up to four cycles per session, before participants met all critical actions and were moved to the classroom for a structured didactic and debriefing session. At the end of the simulation session, participants filled out an online survey to assess their experience.

Results

Since July 2020, our Pediatric Simulation Program has conducted 13 high-fidelity simulations in our simulation center with the split patient model and RCDP. The total number of participants included 50 pediatric residents and one medical student. We reviewed our post-simulation surveys and noted positive feedback in greater than 90% of all participants' responses, who either agreed or strongly agreed that this method taught well the core competencies, as described by the American College of Graduate Medical Education (ACGME) [14].

## Discussion

The split patient method has several advantages. First, the split patient model allows for a modified method of practicing BLS and PALS while reinforcing CRM skills in a high-fidelity environment. This controlled environment can allow for video/audio recording and playback for debriefing as well as data collection for research. Additionally, the simulation session was conducted with RCDP, which allows learners to fix errors, improve resuscitation skills, and apply directive feedback in subsequent cycles [10,15]. The work stations also serve as individual task training for the airway, breathing, and circulation components of BLS and PALS, specifically providing real-time feedback for the participant providing a focus on psychomotor skills, e.g., chest compressions. Finally, the split patient method helps to maintain the social distancing requirements to provide a safe and effective learning environment for the participants as well as the facilitator.

The split patient model has several limitations. First, simulation fidelity is still further decreased, having separated stations as opposed to traditional simulation setups where participants worked closely on the same mannequin detracts from real-world settings. This may be a distracting element, though we were encouraged by participants’ feedback that this cognitive gap was quickly overcome through the repeated cycles in the simulation session. Additionally, only four participants were allowed in the simulation suite, one of which included the code team leader, leaving three team members to address the “ABCs” of initial evaluation and intervention. This concern was addressed in the pre-brief session, where the facilitator informed the residents that they would be acting as the RRT, which routinely has approximately the same number of providers responding to actual RRT calls at our institution. Finally, this method cannot be easily translated to an in situ model, given different COVID restrictions on the wards and intensive care units, wherein space is a valued resource. Nevertheless, this could serve as an active area for future research.

## Conclusions

The split patient model appears to be a viable and safe option for educators to teach SBME for pediatric residents in a high-fidelity environment using RCDP while adhering to social distancing requirements during the COVID-19 pandemic. Participants showed positive feedback in their experience using this method. Future work could include implementation in other fields, such as internal and emergency medicine.
